# Update on the hyper immunoglobulin M syndromes

**DOI:** 10.1111/j.1365-2141.2010.08077.x

**Published:** 2010-02-23

**Authors:** E Graham Davies, Adrian J Thrasher

**Affiliations:** Centre for Immunodeficiency, Institute of Child HealthLondon, UK

**Keywords:** immunodeficiency, hyper immunoglobulin M syndromes (HIGM), class switch recombination defect, CD40 ligand deficiency, activation induced cytidine deaminase (AID)

## Abstract

The Hyper-immunoglobulin M syndromes (HIGM) are a heterogeneous group of genetic disorders resulting in defects of immunoglobulin class switch recombination (CSR), with or without defects of somatic hypermutation (SHM). They can be classified as defects of signalling through CD40 causing both a humoral immunodeficiency and a susceptibility to opportunistic infections, or intrinsic defects in B cells of the mechanism of CSR resulting in a pure humoral immunodeficiency. A HIGM picture can also be seen as part of generalized defects of DNA repair and in antibody deficiency syndromes, such as common variable immunodeficiency. CD40 signalling defects may require corrective therapy with bone marrow transplantation. Gene therapy, a potential curative approach in the future, currently remains a distant prospect. Those with a defective CSR mechanism generally do well on immunologoblulin replacement therapy. Complications may include autoimmunity, lymphoid hyperplasia and, in some cases, a predisposition to lymphoid malignancy.

The Hyper-immunoglobulin M (HIGM) syndromes are a group of primary immunodeficiency disorders in which defective immunoglobulin (Ig) class switch recombination (CSR) leads to deficiency of IgG, IgA and IgE with preserved or elevated levels of IgM. A number of different gene products are involved in this process and defects of a number of these have now been described (Lee *et al*, 2005). Studies of patients affected by these conditions have helped elucidate the process of CSR and the related process of somatic hypermutation (SHM). Most, but not all, patients with CSR defects also show defective SHM. The genetic disorders can be broadly classified into defects restricted to B cells and defects that additionally affect the functions of other cells, including monocytes, macrophages and dendritic cells, whose function requires signalling through the CD40 receptor. The former cause a pure humoral immunodeficiency while the latter are associated with an additional defect of cell-mediated immunity and a consequent susceptibility to opportunistic infections.

In addition to the classical forms of HIGM, other more complex defects of the DNA repair mechanism can also lead to a HIGM-like immunological pattern as part of a more generalized disorder. Additionally, other antibody deficiency disorders, such as common variable immunodeficiency (CVID) or occasionally X-linked agammaglobulinaemia, can present with a picture of low IgG and IgA with preserved IgM thus mimicking HIGM.

A secondary HIGM pattern of immunodeficiency can be seen with congenital rubella infection, malignancy or in patients on antiepileptic medication. This review will not address these forms of the disorder.

An understanding of the details of B cell development and the generation of diverse antibodies of different isotypes is helpful in explaining the different causes of HIGM and will be described here.

## B cell development

**Fig 1:**
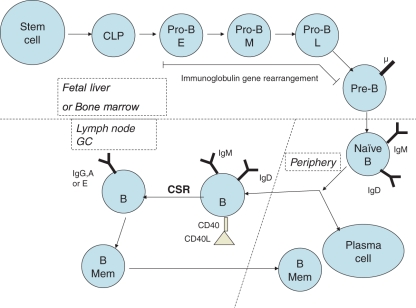
Stages of B cell development. CLP, Common Lymphoid Precursor; Pro B E/M/L, Precursor B cell early/mid/late; B Mem, Memory B cell; CSR, Class switch recombination.

Maturation from the common lymphoid precursor to a class-switched immunoglobulin-producing B cell or a terminally differentiated plasma cell involves antigen-independent and -dependent phases (Fig 1). This has been described in previous reviews (Ghia *et al*, 1998; LeBien, 1998). The antigen-independent phase occurs in the liver during fetal life and thereafter in the bone marrow. *IG* gene rearrangement of the germline DNA to produce unique antibody specificities commences at the pro (precursor)- B cell stage and is completed in the pre- B cell stage. The process of Ig gene rearrangement is initiated by the recombination activating genes (*RAG1* and *RAG2*), which bind to specific recombination signal sequences to initiate double stranded (ds) DNA breaks. There is excision of intervening DNA to bring the required genes into juxtaposition followed by dsDNA repair using the non homologous end-joining (NHEJ) apparatus. Genetic defects in *RAG* genes or in the genes encoding proteins involved in the NHEJ dsDNA repair process (for example Artemis or Ligase IV) result in a failure to generate T and B cell receptors and a clinical picture of severe combined immunodeficiency rather than HIGM (de Villartay, 2009). Exceptions to this are Ataxia–telangiectasia and Nijmegen breakage syndrome, both affecting NHEJ, and sometimes resulting in a HIGM picture (see below).

Immunoglobulin heavy chain gene (*IGH*) rearrangement always results initially in the association of VDJ sequences with the μ chain constant region gene, *IGHM*. Mature naïve B cells express surface IgM and IgD.

## Class switch recombination

**Fig 2:**
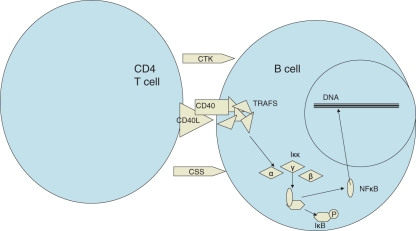
B cell activation to induce CSR. CTK, Cytokines; CSS, Co-stimulatory signals; TRAFS, Tumour necrosis factor receptor (family) associated factors; Iκκ, IκB kinase complex; P, phosphate. IκB(α) inhibitory protein is normally complexed with NFκB, nuclear factor κB, but upon phosphorylation by Iκκ dissociates allowing NFκB to translocate to the nucleus.

The second antigen-dependent stage of B cell development occurs in the periphery and is continued in the germinal centres of lymphoid tissue (MacLennan, 1994; Rajewsky, 1996). This stage is dependent on a number of signals including antigen engagement of the B cell receptor and co-stimulatory signals through the effects of cytokines and direct interaction with T cells. B cells may progress to become plasma cells or follow a route of germinal centre maturation (including CSR) to become memory B cells which express CD27. CD40Ligand/CD40 interaction promotes germinal centre development of B cells and is an absolute requirement for the initiation of CSR and SHM. This process is illustrated in Fig 2. CD40 is a member of the tumour necrosis factor (TNF) receptor family expressed constitutively on the B cell surface while CD40 ligand (or CD154) is a member of the TNF family, which is transiently expressed on activated CD4-positive T lymphocytes during the immune response. Signalling through CD40 occurs through activation of a family of TNF receptor associated factors (TRAFs) and thence via nuclear factor kappa B (NFκB) signalling to the nucleus.

**Fig 3:**
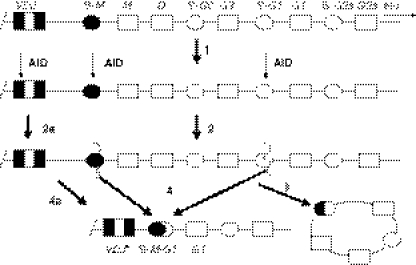
Class switch recombination to generate an IgG1 isotype immunoglobulin. (1) Following initiation of transcription, activation-induced cytidine deaminase (AID) is able to bind to Switch (S) regions and Variable (*IGHV*) genes to initiate class switch recombination and somatic hypermutation. *M, D, G1, G2A* etc. represent constant region heavy chain genes *IGHM….*, etc. S-*M*, S-*G1* etc. represent switch regions of IGH heavy chain genes *IGHM, IGHG2* etc. (2) dsDNA breaks induced in switch regions (see Fig 4). (3) Intervening DNA between switch μ region and target heavy chain constant region switch region (in this case *IGHG1*) is excised. (4) dsDNA repair brings *IGHG1* adjacent to VDJ region. (2a and 4a) Mismatch repair enzymes and error prone DNA polymerases create frequent base substitutions in the *IGHV* genes to create a hypermutated VDJ region VDJ*.

**Fig 4:**
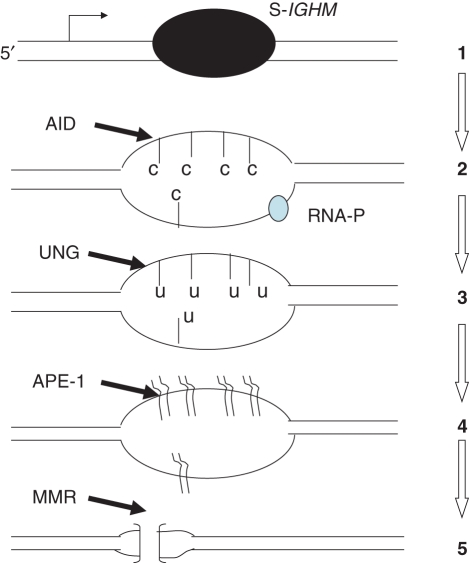
Development of dsDNA breaks in switch regions as part CSR. (1) Transcription is initiated upstream from the *IGHM* switch region (S-*IGHM*). (2) Single strands of DNA available for action of Activation-Induced Cytidine deaminase (AID). RNA-P (ribonucleic acid polymerase) is shown associated with the template strand. (3) Cytidine (C) residues deaminated to uracil (U) by AID. Uracil N Glycosylase (UNG) excises uracil residues. (4) Apurinic/apyridinic endonuclease 1 (APE-1) creates multiple ‘nicks’ in non template DNA strand and a single nick is made in the opposing strand. (5) Mismatch Repair (MMR) enzymes convert single strand ‘nicks’ to a dsDNA break.

CSR involves relocating the previously constructed unique *V(D)J* combination from its association with the constant region gene *IGHM* of IgM to an alternative constant region gene, one of the *IGHG* genes for IgG, *IGHGA* gene for IgA or *IGHE* for IgE (Coffman *et al*, 1993). The process, illustrated in Figs 3 and 4, involves the creation of dsDNA breaks, excision of the intervening sequences and then dsDNA repair. This process is distinct from that involved in immunoglobulin gene (*VDJ*) rearrangement. Recombination occurs between switch (S) regions that are found flanking each constant region gene at the 5′ end and in the intron between the *VDJ* and *IGHM* sequences. The process is initiated by DNA transcription at a point upstream from the S regions. This creates single strand DNA substrates for the enzyme activation induced cytidine deaminase (AID). Through a process of deamination, AID is able to convert cytidine into uracil residues (Bransteitter *et al*, 2003). The enzyme uracil N glycosylase (UNG) excises the uracil residues facilitating the production of a single-stranded break in the DNA strand by an endonuclease (Rada *et al*, 2002). The mismatch repair (MMR) complex of proteins, including the PMS2 (postmeiotic segregation increased 2) protein, has a probable role in converting single stranded into double strand DNA breaks (Schrader *et al*, 2007; Stavnezer *et al*, 2008) as does the MRE11-RAD50-NBS1(MRN) complex (Larson *et al*, 2005). Following excision of the intervening DNA, repair of the dsDNA is initiated. Ataxia-telangiectasia mutated (ATM) protein kinase is involved in this process (Reina-San-Martin *et al*, 2004) and DNA repair employs the NHEJ machinery (Kotnis *et al*, 2009).

## Somatic hypermutation

The process of SHM is also initiated by the action of AID (Fig 3). SHM results in the generation of very frequent mutations in the *IGHV* genes. B cells expressing those mutated *IGHV* genes that have higher antibody affinity are preferentially selected to proliferate in germinal centres in the presence of antigen loaded follicular dendritic cells and follicular B helper T cells thus achieving affinity maturation of the antibody response (Vinuesa *et al*, 2005). The process of SHM is less well understood than CSR. AID function is critical and dsDNA breaks occur as in CSR. The mismatched repair enzymes and error – prone DNA polymerases are employed to achieve repair with a high rate of base substitutions but the NHEJ machinery is not involved (Schrader *et al*, 1999; Poltoratsky *et al*, 2000).

Table I lists the primary genetic disorders causing HIGM syndrome. Although others have classified Hyper IgM syndromes into Types 1–6, this is not helpful in terms of functional consequences and, though defined in Table I, the classification will not be used in this review.

**Table I:** Genetically defined types of HIGM syndrome.

Defect	Inheritance	Infection susceptibility	Lymphoid Hypertrophy	Autoimmunity	Lymphoma	CSR defect	SHM defect	DNA Repair defect
XHIM-CD40 L deficiency (Type 1 HIGM)	XL	Bacterial, opportunistic	−	Yes	No	Yes	Yes	No
CD40 defect (Type 3 HIGM)	AR	Bacterial, opportunistic	−	Yes	No	Yes	Yes	No
NFκB signalling defects (Type 6 HIGM)	XL/AD	Bacterial, opportunistic	−	Yes	No	Yes	Yes	No
AID deficiency (Type 2 HIGM)	AR	Bacterial	++	Yes	No	Yes	Yes	No
AID C terminal defect	AD	Bacterial	+	?	No	Yes	No	No
UNG deficiency (Type 5 HIGM)	AR	Bacterial	+	?	Probable	Yes	No	No
PMS2 deficiency	AR	Bacterial	?	?	?	Yes	No	No
Complex disorders affecting NHEJ DNA repair (Ataxia-Telangiectasia, Nijmegen breakage syndrome)	AR	Mainly bacterial some opportunistic	−	Yes	Yes	Yes	No	Yes

XL, X linked; AR, autosomal recessive; AD, autosomal dominant. AID, activation-induced cytidine deaminase; UNG, uracil N glycosylase; PMS2, postmeiotic segregation increased 2; NHEJ, non-homologous end joining.

Type 4 HIGM refers to a genetically undefined type.

The relative frequencies of the different causes of HIGM have been reported in a group of 140 patients (130 males) with a susceptibility to infections associated with deficiency of IgG and IgA in combination with normal or elevated circulating levels of IgM (Lee *et al*, 2005). These patients underwent extensive genetic testing. By far the commonest defect found, accounting for 98 (70%) of cases, was X-linked HIGM caused by mutations in the gene encoding CD40 ligand (*CD40LG*). Other identified defects affected AID in 4 (3%), UNG and NFκB in one case each and Bruton tyrosine kinase (the cause of X-linked agammaglobulinaemia) in 3 (2%) cases. No mutations were identified in the *CD40* gene, in *SH2D1A* (a gene associated with X linked lymphoproliferative disease) or in *ICOS* (a gene associated with a rare form of CVID). The thirty-three (24%) patients who did not have identified mutations were thought to include molecularly undefined cases of CVID. Other genes now known to be associated with CVID (see below) were not examined in this study.

## HIGM as part of a combined immunodeficiency

Defects of signalling through the CD40 receptor affect more than just B cell function, because CD40 is also expressed on macrophages/monocytes and dendritic cells and lack of signalling to such cells results in impaired handling of opportunistic pathogens. CD40 is also expressed on platelets and, in the presence of inflammation, on endothelial and epithelial cells. The pathway is involved in platelet activation (Inwald *et al*, 2003) and there is increasing evidence for its role in the generation of atheroclerosis (Engel *et al*, 2009). Clinical problems related to defective signalling in non-immunological cells have not been described.

## CD40 ligand deficiency

The first recognized and by far the commonest form of HIGM Syndrome, accounting for at least 70% of patients with CSR defects is caused by mutations in the gene encoding CD40 ligand (*CD40LG*) (Korthauer *et al*, 1993). CD40 ligand is a 39 kD glycoprotein that is a member of the TNF family. The gene, at chromosome Xq26, encodes the molecule expressed in trimeric form on the cell surface and comprises a CD40 binding domain on the cell surface, a short transmembrane domain and a cytoplasmic tail. Occasional symptomatic female carriers with skewed lyonization have been reported (de Saint Basile *et al*, 1999; Imai *et al*, 2006). Expression of the molecule is very tightly regulated occurring only transiently upon activation of CD4+ve T lymphocytes. Testing for expression of the molecule involves overnight activation of T-cells, typically with phytohaemagglutinin and phorbol myristate acetate followed by flow cytometric analysis. It is important to look for other markers of T-cell activation, such as CD25 or CD69 expression, as controls for the activation process (Gilmour *et al*, 2003). This will confirm the diagnosis in the majority of cases in whom mutations result in a lack of protein expression on the cell surface. In a minority of cases with splice site (Seyama *et al*, 1998a) or cytoplasmic tail mutations (Yong *et al*, 2008a) some, or even normal, surface expression is seen making the diagnosis more difficult to confirm. In the neonatal period immaturity in T-cell responses results in failure of expression of this molecule using standard T-cell activation stimuli. Except in rare cases with some protein expression, there is severely impaired production of IgG and IgA. Around half of the patients have elevated levels of IgM at presentation, the remainder having levels within the normal range (Levy *et al*, 1997). The humoral immunodeficiency results in susceptibility to bacterial infections particularly affecting the respiratory tract. There is no response to protein antigens, though some IgM anti-polysaccharide antibodies, including isohemagglutinins, can be produced. Memory (CD27+ve) B-cells are either absent or present in only very reduced numbers (Agematsu *et al*, 1998).

A second consequence of a lack of CD40 ligand expression involves T-cell interaction with macrophages/monocytes. Expression of CD40 on activated monocytes normally results in interaction with activated CD4 cells expressing CD40 ligand to facilitate the production of T-helper cell type 1 (TH1) cytokines (DeKruyff *et al*, 1997), including interleukin-12 and interferon-gamma, which are important in the normal handling of opportunistic intracellular pathogens including *Pneumocystis jiroveci* (Levy *et al*, 1997; Winkelstein *et al*, 2003), *Cryptosporidium* species (Hayward *et al*, 1997), *Toxoplasma gondii* (Subauste *et al*, 1999) and *Mycobacteria* species (Hayashi *et al*, 1999). In the case of *Cryptosporidium* species it has been shown that ligation of CD40 expressed on inflamed biliary epithelium, using soluble CD40L, has a direct effect in killing the organism even in the absence of effector T-cells (Hayward *et al*, 1997).

## CD40 deficiency

This syndrome, a rare cause of HIGM, has been described in patients presenting a very similar clinical picture to boys with X-linked HIGM Syndrome caused by CD40 Ligand deficiency (Ferrari *et al*, 2001; Lougaris *et al*, 2005). Flow cytometric analysis of CD40 expression on B cells and mutation analysis can be used to confirm the diagnosis.

## HIGM syndrome associated with ectodermal dysplasia and immunodeficiency

Signalling through CD40 on B-cells involves NFκB. Boys with X-linked anhidrotic ectodermal dysplasia and immunodeficiency have hypomorphic mutations in the *IKBKG* gene, coding for a protein IKK – gamma part of a kinase complex involved in releasing NFκB from its association with the inhibitory complex IκB allowing translocation to the nucleus. (Zonana *et al*, 2000; Doffinger *et al*, 2001). An overlapping clinical syndrome with autosomal dominant inheritance causing ectodermal dysplasia and immunodeficiency is caused by mutations in *NFKBIA* encoding IκBα, part of the inhibitory complex (Courtois *et al*, 2003). Both these syndromes are very variable both in immunological and non-immunological features. A HIGM pattern of immunodeficiency can be seen with some mutations (Jain *et al*, 2001; Orange *et al*, 2005). Given that NF κB is involved in a number of T-cell and Toll receptor signalling pathways the immunodeficiency is more extensive than simply a class switch defect. Patients are therefore prone to a variety of bacterial and opportunistic infections. The non-haemopoeitic features of the syndrome reflect the usage of NFκB signalling by other cell lineages.

### Clinical complications

Most of the experience with these disorders is from CD40 Ligand deficiency (Levy *et al*, 1997; Winkelstein *et al*, 2003) as the number of reported cases with the other two disorders is too small to allow firm conclusions. Clinical problems occur early in life with a median age at diagnosis of <12 months.

#### Bacterial infections

Recurrent sinopulmonary infections are a consequence of the humoral immunodeficiency in this syndrome. The picture is similar to that seen in other forms of humoral immunodeficiency with recurrent respiratory tract infections potentially leading to bronchiectasis, sinus infections and ear infections. Treatment with immunoglobulin replacement in adequate doses will largely prevent these complications provided the treatment is started before significant damage to the lungs has been sustained.

#### Opportunistic infections

Pneumonia due to *Pneumocystis jiroveci* (PCP) is a presenting feature of this syndrome in around 40% of cases (Levy *et al*, 1997; Winkelstein *et al*, 2003). In the presence of normal T lymphocyte counts and a negative human immunodeficiency virus test, this will be the most likely underlying diagnosis in male infants presenting with PCP. Chronic cryptosporidial infection is another common infection in these patients. Symptomatic chronic intestinal cryptosporidiosis may occur, leading to failure to thrive and weight loss with persistent diarrhoea. Molecular studies for cryptosporidium infection, involving polymerase chain reaction amplication of parasite DNA in patients with CD40 ligand deficiency suggest that subclinical infection is common and in many cases the organism is not detectable by stool microscopy, but only by molecular testing (McLauchlin *et al*, 2003).

Cholangiopathy, with the organism found in the biliary tree, is a common complication of both clinical and subclinical infection. It can result in disturbed liver function tests typically with raised gamma glutamyl transferase levels and, over a period of time, the development of sclerosing cholangitis potentially leading to cirrhosis with a risk of cholangiocarcinoma (Hayward *et al*, 1997; Rodrigues *et al*, 2004). In early series not treated with bone marrow transplantation, chronic liver disease was a feature in 50% of affected individuals and was responsible for early death in many cases (Levy *et al*, 1997). Liver transplantation has been attempted, but with poor results and recurrence of the disease in the transplanted liver. A single successful case of combined liver and bone marrow transplant action has been reported (Hadzic *et al*, 2000). The handling of certain other pathogens requires similar mechanisms to that for cryptosporidium (Subauste *et al*, 1999). Cerebral toxoplasmosis (Leiva *et al*, 1998; Yong *et al*, 2008a) and cryptococcosis (Simon *et al*, 2005) have been described. Cotrimoxazole has been shown to have some beneficial effect in the prevention of toxoplasma infection in immunocompromised individuals but is not completely efficacious (Bucher *et al*, 1997; Weigel *et al*, 1997; de Medeiros *et al*, 2001).

Though CD40/CD40 ligand interaction is thought to be important in the handling of mycobacteria, in practice, tuberculosis is relatively uncommon, being reported in only one case in the two large series (Levy *et al*, 1997; Winkelstein *et al*, 2003) and in occasional other case reports (Shah, 2005). Histoplasmosis was reported in one case in the North American series (Winkelstein *et al*, 2003). Disseminated atypical mycobacterial or Bacillus Calmette-Guérin (BCG) infection has not been reported in patients with CD40 ligand deficiency. However, atypical mycobacterial disease is a relatively common manifestation in defects of NF κB signalling (Dai *et al*, 2004). This may reflect deficiencies of signalling pathways other than through CD40.

Handling of cytomegalovirus (CMV) infection can be problematic in these patients (Levy *et al*, 1997; Winkelstein *et al*, 2003) and disseminated infection can be seen as an initial presenting illness (Benesch *et al*, 2000). CMV has also been implicated in some cases of chronic sclerosing cholangitis (Hayward *et al*, 1997). Human *Parvovirus* infection was described in three cases with leaky splice mutations resulting in partial molecular expression and therefore late presentation of the disorder. In all cases there was a chronic anaemia, which resolved upon commencement of immunoglobulin therapy (Seyama *et al*, 1998b).

#### Neutropenia

Neutropenia is a common complication in boys with CD40 Ligand deficiency. In one series it was reported as occurring at some stage in 50% of cases (Levy *et al*, 1997). The clinical course of the neutropenia may be transient or it may be prolonged and persistent. The precise mechanism by which this occurs is not well understood. Anti-neutrophil antibodies cannot be detected. Early myeloid progenitors express CD40 and ligation has been shown to stimulate myelopoeisis, suggesting that lack of a CD40-mediated stimulation of precursors may play a role (Atkinson *et al*, 1998; Brown *et al*, 1998; Solanilla *et al*, 2000).

Early reports suggested that treatment with high doses of immunoglobulin helped resolve the neutropenia (Banatvala *et al*, 1994) but in the wider European experience the problem only responded to this treatment in around half the cases (Levy *et al*, 1997). The neutropenia is usually responsive to granulocyte colony-stimulating factor.

#### Autoimmunity

Autoimmune complications are relatively common in patients with defects of CD40 signalling. Mature naïve B-cells from CD40 ligand-deficient patients were shown to express a high proportion of auto-reactive antibodies suggesting a role for CD40 ligand/CD40 interaction in mediating peripheral B-cell tolerance (Herve *et al*, 2007). In the Levy study, seronegative arthritis affected 11% and inflammatory bowel disease affected 6% of cases while there were three patients with thrombocytopaenia and one with autoimmune haemolytic anaemia. A small number of cases were also shown to have a variety of autoantibodies though not associated with disease at the time (Levy *et al*, 1997). In the North American series, 12 of 79 (15%) of patients had anaemia. Three of these were due to parvovirus infection. Some of the remainder may have been autoimmune in nature though insufficient detail was reported to be sure (Winkelstein *et al*, 2003). Other occasional cases of autoimmune disease have been reported in CD40 ligand deficiency (Schuster *et al*, 2005).

Autoimmune/inflammatory disease is also a feature in those patients with defective NF κB signalling presenting with a Crohns-like inflammatory colitis (Orange *et al*, 2005).

#### Malignancy

Boys with CD40 ligand deficiency suffer an excess risk of malignant disease affecting the biliary tree (Hayward *et al*, 1997; Levy *et al*, 1997) and intestine, including neuroendocrine tumours (Zirkin *et al*, 1996; Malhotra & Li, 2008). An excess risk of lymphoid malignancy has not been reported.

### Management

Immunoglobulin replacement therapy should be initiated on diagnosis and will largely correct the clinical consequences of humoral immunodeficiency. The susceptibility to opportunistic infections is more problematic. Prophylaxis against pneumocystis should be commenced and consideration given to approaches to correct the underlying disorder.

Administration of recombinant soluble CD40L (Mazzei *et al*, 1995) is a theoretical possibility for corrective therapy but the potential clinical problems associated with unregulated ligation of CD40, not only on immunological cells but also on other cell lineages, mitigates strongly against this approach.

The mainstay of corrective therapy is bone marrow transplantation and this has been successfully employed to treat all three of these conditions. The largest reported series of bone marrow transplantation for CD40 ligand deficiency (Gennery *et al*, 2004) looked at 38 patients from eight European countries. These included a mixture of patients with and without organ damaging complications including liver disease and bronchiectasis. Twenty-six (68%) of the patients survived but four (10%) had autologous reconstitution, one of whom achieved full engraftment after a second procedure. Two of the four with autologous reconstitution had received full and two reduced intensity conditioning (RIC). One patient had extremely poor immunological reconstitution despite achieving full donor engraftment. Overall, a cure was achieved in 22 (58%). Though the cure rate was better in those without liver disease (72%), absence of pre-existing liver disease was not a significant predictor of survival. The presence of lung disease and the use of a mismatched unrelated donor did correlate with a poorer chance of survival. Fully matched unrelated donors did as well as matched sibling donors. RIC regimens were used in too few patients in the survey to draw any conclusions about its usefulness. Other studies have used RIC to good effect but only in small numbers (Kikuta *et al*, 2006). RIC may improve the outcome but carries the risk of rejection, which was not insignificant in the larger survey. Infection was a major factor in all 12 fatal cases (32%). In 6 this was caused by cryptosporidium. There was no association between transplant variables, such as donor type or conditioning used, and the occurrence of cryposporidial infection. Another report indicates that cryptosporidial infection can reactivate after BMT even when apparently subclinical and with negative stools on conventional testing (McLauchlin *et al*, 2003). In the same report it was shown that, despite the fact that antimicrobial treatment for cryptosporidium is poorly efficacious, some patients with cryptosporidial reactivation after transplantation can control the infection and survive. In survivors who had pre-existing liver disease, there is resolution of symptoms and normalization of abnormal liver function tests (authors’ unpublished observations and Dimicoli *et al* (2003).

Given the potential problems with stem cell transplantation, an alternative approach for boys with CD40 ligand deficiency is to adopt a waiting brief; treating with immunoglobulin and cotrimoxazole and monitoring closely for complications such as liver disease and neutropenia. In this approach, transplantation is only performed at the first sign of problems. This is particularly relevant if a fully matched donor cannot be found or if the patient has a hypomorphic mutation. Some boys with the condition will remain well and thrive for a number of years on this regimen (authors’ unpublished observations) Careful attention to avoidance of cryptosporidium exposure is important. The advice given to patients is shown in Table II.

**Table II:** Reducing the risk of cryptosporidium infection.

All drinking water to be boiled or filtered through a professionally fitted filter with <1 μm pore size
Avoid toddler swimming pools and swimming in ponds and lakes
Swimming is allowed in pools from an age >5 years
Avoid contact with farm animals (especially lambs and calves)
Minimize contact with kittens and puppies
Investigate the cause of all diarrhoeal episodes

The numbers of patients transplanted for CD40 deficiency and NF κB signalling defects are too small to derive firm conclusions about this approach in these disorders. Case reports describing successful outcomes are described for both (Dupuis-Girod *et al*, 2006; Mazzolari *et al*, 2007; Tono *et al*, 2007). A recent review suggested that in transplantation for NFκB signalling defects, achieving good levels of engraftment may be difficult(Fish *et al*, 2009) while in another report transplantation corrected the immunodeficiency but failed to correct the colitis (Pai *et al*, 2008).

Gene therapy for CD40 ligand deficiency is under development. However, experiments on CD40 ligand knockout mice show that introduction of *CD40LG,* resulting in constitutive expression of this molecule, caused lymphoproliferative disease in the majority of mice treated that was unrelated to potential insertional mutagenesis (Brown *et al*, 1998; Sacco *et al*, 2000). This indicates that tight control of the expression of this molecule is essential. As a result strategies for developing gene therapy for this condition will need to employ transduction not only of the structural gene but also the elements for regulating its expression, or possibly use methods utilizing DNA or RNA repair (Tahara *et al*, 2004).

## Forms of HIGM syndrome associated with a pure humoral immune defect

Intrinsic B cell defects in the mechanism of CSR result in HIGM syndrome with a pure humoral immunodeficiency without susceptibility to opportunistic infections.

### Activation induce cytidine deaminase (AID) deficiency HIGM

This was the second recognized genetic cause of HIGM syndrome and the first autosomal recessive variety (Revy *et al*, 2000). It is much rarer than CD40 ligand deficiency. AID is expressed transiently and selectively in germinal centre B-cells following stimulation through CD40 and cytokines. It is responsible for deaminating cytidine into uracil residues in the early phase of CSR and SHM. In most cases mutations in the AID gene (*AICDA*) cause HIGM syndrome in an autosomal recessive manner. In the largest reported study, 15 different mutations were reported in 29 patients with no evidence of a genotype phenotype correlation in that study (Quartier *et al*, 2004). Other reports suggest there may be some genotype/phenotype correlation in that patients with mutations in the C terminal part of the gene have impaired CSR but not SHM (Ta *et al*, 2003) and patients with a specific heterozygous mutation in the C terminal end of the molecule exhibit a similar phenotype with autosomal dominant inheritance – see below. In other studies a few commonly occurring mutations were found (Minegishi *et al*, 2000; Zhu *et al*, 2003). In typical AID defects, both CSR and SHM are defective. IgM levels are normal or high while absent or very low levels of IgG, IgA and IgE are seen. Memory B cells expressing CD27 are present in normal numbers.

### AID C terminal defect

An autosomal dominant form of AID deficiency has been described as caused by a missense mutation in the C terminal domain of the molecule, which is the domain involved in nuclear egress. Studies on these patients showed that there was defective CSR but not SHM, suggesting that the C terminal part of the molecule is involved with the enzyme complexes involved in DNA repair in CSR but not repair after SHM which uses different mechanisms (Imai *et al*, 2005).

### Uracil N glycosylase (UNG) deficiency

Deficiency of this enzyme has been described as another cause of HIGM syndrome in a small number of patients (Imai *et al*, 2003a). Patients suffer a similar clinical picture to AID deficiency. CSR but not SHM is impaired in this disorder although there is marked skewing of the bases involved in SHM towards G–C rather than A-T. Numbers of cases reported are too small to draw firm conclusions about the clinical phenotype but frequent bacterial infections and lymphoid hypertrophy seem to occur.

### PMS2 deficiency

PMS2 (postmeiotic segregation increased 2) is one of the proteins involved in the complex mediating mismatch repair of DNA. Along with other members of the mismatch repair enzyme complex, mutations in *PMS2* have been identified as being associated with gastrointestinal adenocarcinomas (Gologan & Sepulveda, 2005). A recent report described the presence of mutations in *PMS2* in three patients with a defect in DNA cleavage as part of a CSR defect (Peron *et al*, 2008). There was a partial immunological phenotype of HIGM with low (but not absent) IgG associated with complete IgA deficiency in one patient and a low IgA in one other which corrected over time. CSR was markedly abnormal *in vitro.* There was either no defect or only a mild defect of SHM. The patient with the most severe immunophenotype was reported as having severe bacterial infections prior to diagnosis and subsequently developed colonic adenocarcinoma.

### Other undefined forms of HIGM syndrome due to defects of CSR mechanism

Not all cases of HIGM syndrome can be ascribed to known genetic defects. The characteristics of two defects of CSR both inherited in an autosomal recessive fashion but for which no genetic cause has yet been identified have been reported. In the first (Imai *et al*, 2003b) a B cell defect affecting CSR downstream of DNA transcription step was found without radiosensitivity. SHM was not affected. A possible cofactor for AID function has been postulated. A second form of CSR abnormality reported in 16 patients was associated with radiosensitivity implying a defect in dsDNA repair mechanisms (Peron *et al*, 2007). Durandy (2009) recently reported an update on these defects.

### Clinical complications

Other than AID deficiency, very small numbers of cases have been reported on which to base a description of clinical features. Some of the defects, such as PMS2, may result in a partial phenotype as far as immunological findings are concerned though clinical features are not fully described. The main potential clinical complications are described below.

#### Infections

In AID deficiency, two case series have been reported (Minegishi *et al*, 2000; Quartier *et al*, 2004). Prior to commencing treatment with immunoglobulin, recurrent severe infections mainly bacterial and most often causing pneumonia were seen. Other sites of infection were the skin, lymph nodes, gastrointestinal tract and central nervous system, the last including bacterial meningitis and one case of *Herpes simplex* encephalitis. The onset of infections was early, usually before 2 years of age, but in the study reported by Minegishi *et al* (2000) a considerable delay often occurred before a diagnosis of immunodeficiency was made. Opportunistic infections were not described. The numbers of cases reported with disorders other than AID deficiency is too small to draw firm conclusions but the clinical susceptibility to bacterial infections seems to be similar.

#### Lymphoid hypertrophy

Marked lymphoid hypertrophy is a clinical feature of AID deficiency that is reported in one half to two-thirds of cases (Minegishi *et al*, 2000; Quartier *et al*, 2004). It has also been described in the other forms of CSR defect (Durandy *et al*, 2006). It can affect all lymphoid tissues but peripheral lymphadenopathy and tonsillar hypertrophy were most commonly reported. Splenomegaly was relatively uncommon, being reported in 2 of 29 in one series (Quartier *et al*, 2004). Lymph node and tonsillar histology characteristically shows giant germinal centres (Revy *et al*, 2000). The driver for such germinal centre hypertrophy is not clear. Interestingly, treatment with immunoglobulin seems to reduce the likelihood of developing this complication with only two of 29 patients developing this complication after commencement of treatment (Quartier *et al*, 2004). In AID knock out mice, Peyer’s patch hypertrophy has been shown to be driven by intestinal bacterial overgrowth (Fagarasan *et al*, 2002).

#### Autoimmunity

In both reported series of AID-deficient HIGM syndrome, autoimmune complications were described with an incidence of around 20% and included immune cytopenias, arthritis and hepatitis (Minegishi *et al*, 2000; Quartier *et al*, 2004). Potential mechanisms for the autoimmune process have been reviewed (Jesus *et al*, 2008). It has also been postulated that the B cell lymphoproliferation characteristic of the condition leads to the development of ectopic lymphoid tissue in non lymphoid organs, predisposing to organ specific autoimmunity (Hase *et al*, 2008). Autoimmune complications would also be expected to occur in UNG-deficient forms of the disorder.

#### Malignancy

To date, despite the tendency to lymphoid hyperplasia in AID deficient patients, malignant lymphoproliferation has not been described. There is a probable predisposition to B cell malignancy in UNG deficiency although this has not been reported in the few cases described (Durandy *et al*, 2006). UNG knock out mice are prone to B cell lymphomas consistent with a role for the base excision function of this enzyme in correcting mutagenic influences (Nilsen *et al*, 2003).

### Management

The mainstay of treatment for these forms of HIGM syndrome is immunoglobulin replacement therapy. This is reported as reducing markedly the incidence of bacterial infections and also reducing the likelihood of developing lymphoid hypertrophy (Quartier *et al*, 2004). Early diagnosis and initiation of treatment is important in reducing the likelihood of the patient developing bronchiectasis and/or chronic sinusitis. Studies of immunoglobulin-deficient patients generally have found that these complications are usually established before initiation of replacement therapy and may then progress despite treatment (Wood *et al*, 2007). Subcutaneous treatment with immunoglobulin has been shown to be both efficacious and acceptable to antibody-deficient patients (Chapel *et al*, 2000).

Autoimmune complications are generally managed along the lines used in non-immunodeficient patients. The authors are not aware of any reports of usage of anti CD20 monoclonal antibody (rituximab) in these disorders.

Corrective therapy, such as bone marrow transplantation, cannot generally be justified given the fact that these are pure humoral deficiencies showing good response to immunoglobulin therapy. Theoretically, such an approach might be justified in patients with uncontrollable autoimmune manifestations or in those who have developed lymphoid malignancies.

## Forms of HIGM syndromes associated with syndromes affecting DNA repair

Ataxia- telangiectasia (A-T) and Nijmegen Breakage syndrome involving defects in ATM and NBS1, respectively, are both conditions in which immunodeficiency can be a prominent feature (Taalman *et al*, 1989; Staples *et al*, 2008). These enzymes are closely involved in CSR (Reina-San-Martin *et al*, 2004; Kracker *et al*, 2005). ATM deficiency does not affect SHM while NBS1 does (Pan-Hammarstrom *et al*, 2003). Clinically, a number of A-T patients have been described as presenting with a classical HIGM pattern of immunoglobulin deficiency (Etzioni *et al*, 2007; Soresina *et al*, 2008; Noordzij *et al*, 2009). More commonly there is IgA and/or IgG2 deficiency. Since the *IGH* heavy chain genes for these two isotypes are amongst the furthest downstream from the VDJ genes it has been postulated that the defective CSR function in A-T is more marked for rearrangements involving longer intervening DNA sequences (Giovannetti *et al*, 2002). In both conditions chromosomal translocations, particularly affecting the immunoglobulin and T cell receptor genes on chromosomes 7 and 14, are commonly found. Lymphomas are common in both conditions. In A-T these are more often of T rather than B cell origin. Ataxia-telangiectasia-like disorder (ATLD) is caused by mutations in the *MRE11A* gene, also involved in CSR and SHM. Though defective CSR has been shown, clinical or laboratory evidence for immunodeficiency is usually not present (Delia *et al*, 2004; Taylor *et al*, 2004; Fernet *et al*, 2005).

## HIGM as part of other primary antibody deficiency disorders

Preserved levels of IgM may be found at presentation in patients presenting with antibody deficiency syndromes not due to any of the classical HIGM disorders. This includes X-linked agammaglobulinaemia caused by mutations in Bruton tyrosine kinase (*BTK*). In a series including 140 patients with a HIGM picture, *BTK* mutations were found in three patients (Lee *et al*, 2005). In the same study, 33 patients without an identifiable genetic defect were thought to include patients with CVID. This disorder is often part of the differential diagnosis in patients with a HIGM as preservation of IgM production is not unusual, at least in the early stages of CVID. Sometimes a frank HIGM picture is seen. Causes of CVID have recently been reviewed (Yong *et al*, 2008b). A number of genetic defects have been described, which may account for around 10% of cases, with genetic lesions still to be identified in the remainder. Mutations in genes encoding TACI (*TNFRSF13B*), ICOS (*ICOS*), CD19 (*CD19*), and, most recently, B cell activating factor receptor (BAFF-R; *TNFRSF13C*) (Warnatz *et al*, 2009) all involved in the process of B cell activation, have been described in CVID. It can be postulated that failure of signals through these receptors could affect the antigen-dependent pathway of B cell maturation during which CSR and SHM take place. Recently, variant sequences in MSH5, one of the complex of mismatch repair enzymes involved in CSR and SHM have been shown to be associated with some cases of CVID and IgA deficiency (Sekine *et al*, 2007). This finding suggests that defects in the mechanisms of CSR (and thus true HIGM disorders) may account for a proportion of genetically undefined cases labelled as having CVID.

## Conclusion

Studies on patients with HIGM syndrome can now identify the genetic cause in around 75–80% of cases. The remainder is currently undiagnosed at the genetic level. Treatment options depend on the type of defect with those involving defective CD40 signalling requiring consideration of corrective therapy whilst those with intrinsic B cell defects mostly require immunoglobulin replacement therapy alone.
